# VATS decortication for trapped lung in malignant pleural effusion: results of surgery

**DOI:** 10.1186/1749-8090-8-S1-O229

**Published:** 2013-09-11

**Authors:** T Minchev, E Manolov, V Marinchev, A Angelov, I Stoimenov, B Penev

**Affiliations:** 1 Thoracic Surgery, Tokuda Hospital, Sofia, Bulgaria; 2 ICU, Tokuda Hospital, Sofia, Bulgaria; 3 Medical Imaging, Tokuda Hospital, Sofia, Bulgaria

## Background

Surgery can only offer palliation in an attempt to slow the progression of malignant pleural effusion (MPE). We want to assess the effectiveness and safety of decortication in selected patient with trapped lung compare with control group with talk pleurodesis only.

## Methods

We reviewed our VATS decortication results in 26 patients with MPE over a 6 year period, compare with 40 patient treated with talk pleurodesis and drainage only. Major symptoms were chest pain, cough and dyspnea, and radiographic findings of pleural fluid. Trapped lung was demonstrated after MPE evacuation in all patients preoperatively. Patient with malignant mesothelioma end bronchial carcinoma and atelectasis was excluded from this study.

## Results

The patients underwent subtotal (80%) or total decortication (20%). No surgical mortality and the morbidity rate was 3%. Morbidity included prolonged air leak (n = 2), reaccumulation of pleural fluid (n =1). Palliative results included dyspnea and cough relief in all patients with VATS decortication, chest relief in (85%) and pleural fluid control in 23 (96%) patients. Median survival was 18 months in VATS patients compare to 6 mounts in patient with talk pleurodesis only. Chi test was used to compere groups and Kaplan-Mayer method for estimating the survival analysis.

## Conclusions

We conclude that VATS decortication and talk pleurodesis safely provides effective treatment of pleural effusion and symptoms and therefore excellent palliation in selected patients with malignant pleural effusion and trapped lung.

